# Access to Healthy Wheat and Maize Processed Foods in Mexico City: Comparisons across Socioeconomic Areas and Store Types

**DOI:** 10.3390/nu14061173

**Published:** 2022-03-10

**Authors:** Ana Cecilia Fernández-Gaxiola, Carlos Cruz-Casarrubias, Selene Pacheco-Miranda, Joaquín Alejandro Marrón-Ponce, Amado David Quezada, Armando García-Guerra, Jason Donovan

**Affiliations:** 1 Centro de Investigación en Nutrición y Salud, Instituto Nacional de Salud Pública (INSP), Universidad No. 655, Colonia Santa María Ahuacatitlán Cerrada Los Pinos y Caminera, Cuernavaca C.P. 62100, Mexico; anafdezg@hotmail.com (A.C.F.-G.); casarrubiasnt@gmail.com (C.C.-C.); selene.86517@gmail.com (S.P.-M.); joaquin.marron@hotmail.com (J.A.M.-P.); 2 Centro de Investigación en Evaluación y Encuestas, Instituto Nacional de Salud Pública (INSP), Universidad No. 655, Colonia Santa María Ahuacatitlán Cerrada Los Pinos y Caminera, Cuernavaca C.P. 62100, Mexico; amado.quezada@insp.mx; 3 International Maize and Wheat Improvement Center (CIMMYT), Carretera México-Veracruz Km 45, El Batán, Texcoco C.P. 56237, Mexico; j.donovan@cgiar.org

**Keywords:** food environment, Nutri-Score, food retail, health and nutrition claims, food prices, Latin America

## Abstract

The contributions of processed foods to the overweight and obesity problem in Latin America are well known. Engagement with the private and public sectors on possible solutions requires deeper insights into where and how these products are sold and the related implications for diet quality. This article characterizes the diversity of wheat and maize processed foods (WMPFs) available to consumers in Mexico City. Data were gathered across nine product categories at different points of sale (supermarkets, small grocery stores, convenience stores) in high and low socioeconomic (SE) areas. We assessed WMPFs based on Nutri-Score profile, price, and health and nutrition claims. Roughly 17.4% of the WMPFs were considered healthy, of which 62.2% were pastas and breads. Availability of healthy WMPFs was scarce in most stores, particularly in convenience stores Compared to supermarkets in the low SE area, those in the high SE area exhibited greater variety in access to healthy WMPFs across all product categories. In the low SE area, healthy WMPFs were priced 16–69% lower than unhealthy WMPFs across product categories. The extensive variety of unhealthy WMPFs, the limited stock of healthy WMPFs in most retail outlets, and the confusing health and nutrition claims on packaging make it difficult for urban consumers to find and choose healthy WMPFs.

## 1. Introduction

Food processors and food retailers target specific products to specific subgroups of consumers in response to heterogeneity in economic, demographic, and behavioral factors. In providing food products tailored to meet consumer demands [1,2,3], segmentation strategies potentially deliver higher sales and profits for processors and retailers. In North America and Europe, research has supported these strategies by shedding light on consumer-related trends and issues related to, for example, perceptions on GMO-based foods [4], attitudes towards nutrition [5], food safety and quality (including organic and locally produced) [6], and acceptance of new food and agricultural products [7,8,9]. Discussions have explored potential solutions to address the private sector’s targeting of poorer areas with less-healthy and lower-cost foods, including the promotion of farmers’ markets in lower-income areas [10,11]. However, limited research has examined how the food industry in developing and emerging economies targets different products to consumers with different economic means and the implications of such targeting for access to healthier foods. 

While conceptualized and interpreted in different ways [12], a common feature of food systems and food environments is the strong influence of the food industry on access to and preferences for food. The narratives on private sector engagement in food systems vary, from the private sector being the scaler of technological innovations in food production and processing [13] to its being responsible for “food deserts” in lower-income communities [14]. Turner and colleagues (2018) [15] defined the food environment as the interface where people interact with the wider food system to acquire and consume foods. The authors identified two constructs: a personal domain and an external domain. 

The personal domain “relates to individual level dimensions such as food affordability, accessibility, convenience, and desirability”. 

The external domain considers “the world of opportunities and constraints that are out there within a given context”, which is shaped by price, vendors, food availability and product properties. Various studies have focused on the internal and external domains and how these are shaped by food processors and retailers. Some found that in-store healthy foods were less likely to be found in low-income neighborhoods in cities in the United States [16], Australia [17], Paraguay [18], and Brazil [19]. Food price and the consumers’ preferences, which, in turn, may be shaped by food industry marketing practices [20], are considered to be important factors that influence in-store food selection [21,22]. 

Evidence suggests that maintaining a healthy diet is relatively expensive given the cost of items that comprise a lower quality diet which tends to include more processed, energy-dense foods [23]. Food vendors considered high cost a major barrier to consumers’ willingness to increase their purchases of healthy foods [22]. The type of retail outlet (e.g., supermarkets versus convenience stores) can also shape food availability and food purchases [20]. For example, smaller food distributors are more likely to obtain stocking agreements with independently owned grocery stores for the promotion of less-healthy foods with free-standing displays and discounted products [21]. The nutrient profile of individual foods within a food category can vary greatly, and in broad terms, those with healthier profiles have higher prices. However, in some cases, foods with lower nutritional quality have higher prices, especially among ultraprocessed wheat products (e.g., cereal bars, cereals, and cookies) and fruit juices for example [24]. 

Researchers have called for policies and interventions (e.g., social marketing, sugar taxes, public purchasing programs) to incentivize the consumption of foods of higher nutritional quality [25,26]. Others have called for increased engagement with the food industry on options to promote the consumption of healthier foods. As noted by Haddad and colleagues (2016) [27], “The private sector could help to tilt food systems towards higher-quality diets, and could respond innovatively to targets and regulations. We need better mechanisms for public–private dialogue to shape and implement research priorities.” Regardless of the approach, a more nuanced understanding of market segmentation by the food industry is needed as a starting point for a deeper discussion on the challenges at hand in urban food systems and the different options available for remediation with public and private sectors. 

**Figure 1:**
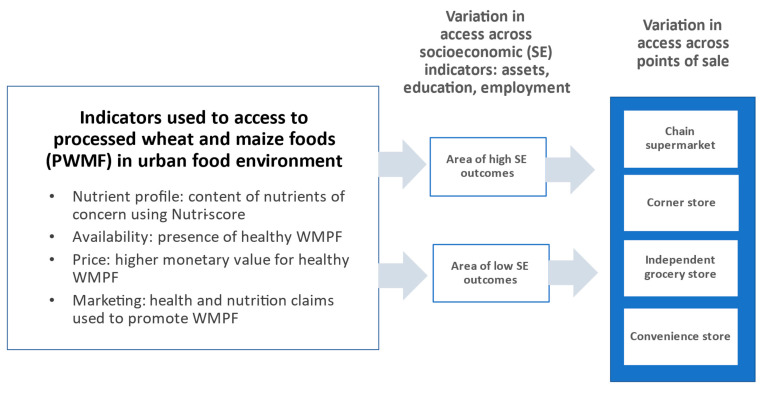
Framework for assessing access to wheat and maize processed foods (WMPFs) in an urban food environment.

This study examines, across different types of retail outlets, access by urban consumers to processed wheat and maize products—major occupiers of food retail shelf space that include crackers and chips, tortillas, breakfast cereals, breads, pastas, and frozen foods. As shown in Figure 1, we consider access in terms of nutrition content, availability, price, and marketing claims, which are explored across different levels of socioeconomic attainment and points of sale. Specifically, we explore access to wheat and maize processed foods (WMPFs) in Mexico City—one of Latin America’s most populated cities, with high income inequality levels [28] and sophisticated food processing and retail sectors [29,30]. The overall increased availability of low-cost, nutrient-dense processed foods has contributed to the rapid increase in obesity occurring in Mexico and elsewhere in Latin America [29]. In Mexico, overweight and obesity are present in 35.6% of school-aged children, 38.4% of adolescents, and 76.8% and 73% of adult women and men [30], and lowering these levels is a major public health priority. Among foods produced in Mexico, WMPFs represent roughly 30.7% of total food processing sector sales [31]. Maize tortillas; maize products (20.6%); and sweet, white, and wheat breads (11.5%) account for 32.1% of total energy intake in the Mexican diet [32]. Ultraprocessed foods based on wheat and maize ingredients (i.e., cookies, pastries, salty snacks, and ready-to-eat cereals) comprise a major component of the Mexican diet (16.2% of total energy intake) [33]. A research group at the National Institute of Public Health of Mexico recently reported that maize tortillas and tortilla products contributed 20.6% of total energy intake in the diet in the Mexican population, while fruits and vegetables, by comparison, contributed only 5.6% [32]. 

This study seeks insights into the following two questions: (1) What are the characteristics of WMPFs in terms of nutritional quality, health and nutrition claims, and price? (2) How do these vary by levels of socioeconomic attainment and points of sale? The findings here will allow for insights on the nutritional challenge and possible entry points for future engagement with public and private sectors on potential options for promoting increased access to healthy WMPFs for different types of urban consumers.

## 2. Materials and Methods

### 2.1. Study Design and Sampling

This cross-sectional study presents data on access to WMPFs collected in Mexico City and its surrounding area between March and June 2019. Principal component analysis was used to characterize “basic geographical areas” (BGAs)—administrative units in Mexico City for which data were available. The analysis included 14 variables on socioeconomic (SE) characteristics: housing materials and access to public utilities (e.g., floor type electricity, running water), material goods (e.g., ownership of televisions and refrigerators), and population characteristics (e.g., years of education, access to health care services, and employment). BGA-level data and information on frequency and location of stores were obtained from the National Statistic Directory of Economic Units and the National Population and Household Census 2010. Those BGAs which did not contain at least two supermarkets and two independently owned grocery stores were excluded. BGAs were categorized into low and high SE BGAs using their first and last deciles of the first principal component of SE characteristics. These criteria resulted in eight high SE areas and eight low SE areas in Mexico City and its surroundings; i.e., the high SE was in the Polanco area, the low SE was in the Chimalhuacán area. Additionally, field reconnaissance of each selected area was conducted to assess diversity in the type of food retailer and ensure the minimum number of food retail outlets, as well as their distribution, for the final selection of the two areas for the study. 

Four types of food retail outlets were considered for each area: chain supermarkets, independently owned grocery stores, convenience stores, and corner stores. Trained personnel visited each store in a selected SE area and included adjacent BGAs if a specific type of food retailer was not accessible to the data collection team. Twenty-seven food retail outlets were confirmed within each SE area. We were able to increase the final sample to 2 convenience stores (which were not originally included), 2 neighborhood corner stores, and 20 independently owned grocery stores in both areas and 3 supermarkets in the high SE area and 2 supermarkets in the low SE area. Most low SE areas did not contain a chain supermarket, thus requiring adjustments in the field to ensure adequate supermarket coverage. 

### 2.2. Data Collection and Management

We distinguished nine categories of WMPFs for inclusion in the study: ready-to-eat foods, breakfast cereals, salty snacks, cookies, flour, breads, bars and pastries, pastas, and maize tortillas and tortilla products. Within each category, we considered only packaged and labeled products, regardless of origin, which listed wheat or maize among the first three ingredients. We excluded nonpackaged products from traditional stores such as bakeries and tortillerias. Data were collected by taking photographs of information available on each product in the selected product categories at the store. A standardized methodology [34] was applied for the assessment of each product where a set of photographs captured the following: front of package, content, serving size, number of servings, bar code, nutritional information, ingredients, health and nutrition claims, and price.

Each product was classified according to its Nutri-Score profile [35]. The score considers calories, nutrients, and ingredients. Based on the score, each food is classified into one of five Nutri-Score profiles (from the healthiest to the least healthy): A (score ≤ −1), B (score 0–2), C (score 3–10), D (score 11–18), and E (score ≥ 19). Nutrients and ingredients that negatively impact health include calories, sugars, saturated fats, and sodium, while those with a positive impact include fruits, vegetables, fiber, and protein. Negative nutrients and ingredients can take 10 points each while positive ones can add 5 points each [35] As a result, the healthiest products should not only contain nutrients and ingredients with a potentially positive impact on health but also be free of nutrients and ingredients with a potentially negative impact on health. The higher extent to which a product contains more nutrients and ingredients with a potentially negative impact on health and/or lacks nutrients and ingredients with a potentially positive impact on health, the more likely it will be classified as “unhealthy” (i.e., group C, D or E). 

Prices obtained from each food category were converted from Mexican pesos to US dollars (USD) by using the exchange rate during the collection period (USD 1 to MXN 19.15) [36]. Prices of each WMPF were scaled by 100 g of product and by package. 

### 2.3. Ethical Considerations

The study protocol and procedures were approved by the Ethics Committee of the International Maize and Wheat Improvement Center (CIMMYT). Consent to conduct in-store data collection was obtained by email or in person from store representatives. 

### 2.4. Statistical Analysis

We described the diversity of access to WMPFs by SE area and the type of store with the percentage of distinct WMPFs that were found in both SE areas, in low SE areas exclusively, or in high SE areas exclusively. The same analysis was used to describe the availability of WMPFs by Nutri-Score and food product categories by SE area including all products in the sample. 

We applied a specified a multiple linear regression model for analyzing price differences between SE areas. The log of price of WMPFs per 100 g was specified as the dependent variable. Explanatory variables included three categorical variables and all their two and three way interactions, these variables were: SE area (low/high), Nutri-Score profile (coded as healthy/unhealthy) and food group. We obtained geometric means for each combination of SE area, Nutri-Score profile, and food group category, as well as geometric mean ratios between SE areas, by exponentiating the corresponding linear combination of model coefficients. We derived 95% confidence intervals by back transforming the confidence limits of the linear combinations. Additionally, we fitted a separate model for each type of retail store with the log of price per package as a dependent variable and the same specification of predictors as previously described and obtained the aforementioned differences by SE area and their confidence intervals. We reported the use of health and nutrition claims as percentages for each combination of food group category, SE area, and Nutri-Score profile, with their corresponding logit-transformed 95% confidence intervals [37], and calculated their differences in percentage points by SE area. Categories with less than 5 observations were excluded from analyses. Robust standard errors from all models were adjusted for data clustering within retail stores [38]. All analyses were performed in Stata (version 14).

## 3. Results

### 3.1. Food Supply and Stock of Healthy WMPFs across Socioeconomic (SE) Areas and Food Retail Outlets

We recorded a total of 7567 WMPFs available in both SE areas, including unique and repeated products. Of these, 2462 were unique WMPFs. Among unique WMPFs, only 28.8% of them were classified as healthy (Table 1). Supermarkets offered the greatest variety of healthy products while convenience stores showed the least (614 vs. 29). However, the number of healthy WMPFs offered in supermarkets and convenience stores varied greatly between SE areas: 79.3% of unique WMPFs in supermarkets were available exclusively in the high SE area whereas 8.6% were available exclusively in the low SE area. In convenience stores, 44.8% of healthy WMPFs were in the high SE area exclusively, 3.5% were in the low SE area exclusively, and 51.7% were in both areas. Cookies, salty snacks, pastas, and bars and pastries were the WMPF categories with the greatest variety (636, 387, 290, and 258 products, respectively). Breakfast cereals, *tortillas* and *tortilla* products, flour, and breads were the WMPF categories with the least variety of products (203, 157, 155, and 154 products, respectively). Independently owned grocery stores provided the most homogeneous set of WMPFs in both low and high SE areas (from 86.1% to 100% similarity within WMPF categories). Across WMPF categories, cereal bars and pastries (38.7%), salty snacks (36.7%), and breakfast cereals (29.1%) were more frequently found in both SE areas. 

### 3.2. Availability of WMPFs across Socioeconomic (SE) Areas by Nutri-Score Profile

Looking across the sample, only 11.7% and 5.6% of available WMPFs were included in the A and B Nutri-Score profiles, respectively. We found considerably greater availability for healthy WMPFs (i.e., Nutri-Score profiles A and B) in the high SE area (Table 2). Indeed, healthy items in the cookies and bars and pastries categories were only available to consumers in the high SE area. Consumers in the low SE area had basically no nearby access to healthy (i.e., included in groups A and B) breads, bars, and pastries. Unhealthy WMPFs (i.e., Nutri-Score profiles C, D, and E) were widely available in both SE areas. 

### 3.3. Price Variation of WMPFs across Socioeconomic (SE) Areas and Retail Outlets

The geometric mean (GM) prices per 100 g and per package of healthy and unhealthy WMPFs food profiles were significantly higher in the high SE area compared to the low SE area (Table 3). For example, GM price per 100 g of healthy cookies was roughly 3-fold in the high SE area compared to the low SE area (GM ratio = 3.01; 95%CI: 2.15, 4.21), for unhealthy cookies GM price per 100 g was about 50% higher in the high SE area compared to the low SE area (GM ratio = 1.53; 95%CI: 1.18, 1.99). Within the low SE area, GM price per 100 g for healthy WMPFs was 16–69% lower compared to GM price per 100 g for unhealthy WMPFs (e.g., USD 0.20 95%CI: 0.018, 0.23; USD 0.64 95%CI: 0.59, 0.71, for total WMPFs, respectively). Supermarkets charged the highest prices for healthy and unhealthy WMPFs per package across all retail outlets and WMPF categories in both SE areas (Table 4). GM prices in supermarkets for healthy and unhealthy WMPFs were 165% (GM ratio: 2.56; 95%CI: 1.41, 4.97) and 86% (GM ratio: 1.86; 95%CI: 1.42, 2.44) more expensive in the high SE area compared to the low SE area. GM price differences for all WMPFs in other food retailers were smaller and most of them not significant between SE areas.

### 3.4. Health and Nutrition Claims Displayed in WMPFs across Socioeconomic (SE) Areas

Health and nutrition claims were found on 23.2% of all WMPFs. They were more frequently found on healthy products in the high SE area compared with the low SE area for cookies (+32.9 p.p., 95%CI: 4.6, 61.3) and maize tortillas and tortilla products (+32.7 p.p., 95%CI: 9.9, 55.6) (Table 5). A considerable proportion of unhealthy WMPFs employed health and nutrition claims, particularly in the high SE area (20.1%, 95%CI: 14.8, 26.5). Unhealthy WMPF categories where health and nutrition claims were used in both SE areas included breakfast cereals (>45%) and flour (>25%). In the high SE area, 40.4% (95%CI: 30.9, 50.5) of unhealthy maize tortillas and tortilla products displayed health and nutrition claims.

## 4. Discussion

In this study, we collected data on the diversity of WMPFs available to consumers in Mexico City paying attention to variation in access by socioeconomic areas, retail outlets, nutrient profile, and health and nutrition claims employed. The study was motivated by the need for data on what WMPFs were accessible to consumers, as an input for future discussions with the private sector on their role in marketing available healthy and nutritious WMPFs for urban consumers. Our findings highlighted various issues in the retail landscape for WMPFs that have important implications on the dietary options available to consumers, especially those in lower-income neighborhoods. 

### 4.1. Most WMPFs Are Not Healthy

Cereals, including wheat and maize, can contribute essential amino acids, minerals, and vitamins and beneficial phytochemicals and dietary fiber components to the human diet. Although wheat and maize have traditionally been processed for human consumption, current WMPFs normally exceeded the daily recommended intake of sodium, saturated fat, trans fats, and added sugars established by WHO to prevent overweight, obesity, and chronic diseases [33,39], and their consumption is associated with lower-quality nutrient profiles [40,41] and often leads to unhealthy diets and overall poorer health conditions [42]. We found a majority and considerably greater variety of unhealthy WMPFs in both SE areas and across all food retail outlets. WHO’s current global plan is a 30% reduction in salt/sodium population intake along with policy measures that engage food retail outlets to improve the availability of healthier products with reduced content of salt/sodium, saturated fats, and free sugars [43]. Our findings showed the urgent need for more progress towards these goals, as only 11.7% and 5.6% of available WMPFs were from the A and B categories of the Nutri-Score in the whole sample. The current need for food systems transformation for improved nutrition and health is alarming [44].

### 4.2. Consumers in Richer Areas Have Access to a Greater Diversity of WMPFs 

The high SE area had much more diversity in terms of WMPFs available as compared to the low SE area. Previous studies had found that in-store healthy foods were less likely to be found in low-income neighborhoods in cities, and our findings were consistent with these [16,17,18,19]. However, in our study, the high SE area had the most diversity of not only in-store healthy WMPFs but also unhealthy ones. As a result, both areas offered limited options for healthy WMPFs, restricted to few WMPF categories. A recent panel survey analysis of purchase and intake data in urban areas in Mexico showed that higher SE groups from urban areas had greater purchases and intakes of less-healthy foods than lower SE households [45]. Our findings are consistent and further suggest that consumers in the high SE area have access to and probably purchase a greater variety of healthy products, but also unhealthy ones, than lower SE households.

Mexico’s 8% sales tax on unhealthy foods resulted in a 5.8% decline in the purchase of taxed foods among households of medium SE status and a 10.2% decline among households of lower SE status [46]; thus, impact on high SE has not been measured or acknowledged as a risk, especially because complex urban surroundings such as those of Mexico City imply people moving around different areas to access their foods, and food supply in high SE areas may be seen as a model to be followed or symbol of status, setting the example of procurement for vendors stocking WMPFs in other SE areas.

### 4.3. Specialization in Retail Stores across Socioeconomic Areas

We found specialization in retail stores, with convenience stores and neighborhood corner stores specializing in WMPFs with Nutri-Score profiles D and E (unhealthy) WMPFs. Supermarkets had the greater stock of products with profiles A and B (healthy groups). In the low SE area, neighborhood corner stores and supermarkets could provide, in theory, access to healthier WMPFs; however, supermarkets are scarce in low SE areas. Similarly, in high SE areas, neighborhood corner stores and supermarkets could provide greater access to healthier WMPFs, but neighborhood corner stores, as well as independently owned grocery stores, tended to be scarce. Yet, increasing in-store healthy products in small food retail outlets is feasible and can be achieved with support and understanding of healthy product criteria [47] or incentives [48]. 

Interventions to improve healthy food offerings in small stores should consider the diverse environments, stocking practices, and supply mechanisms with innovative distribution practices of small stores, particularly nontraditional food retailers [49]. For example, retail outlets in the low SE area could stock cookies, bars and pastries, and breads classified in the healthy categories (A and B groups) and provide consumers access to them.

### 4.4. Higher Prices Prevail for Unhealthy WMPFs

The consumption of healthy food is mostly consistently related to income, contrary to the intake of unhealthy ones [50]. However, we found, in general, that prices per 100 g and per package of unhealthy WMPF products were higher than prices for healthy products in most WMPF categories, but prices per package differed by SE area. By package, healthy products tended to be more expensive, particularly in the high SE area. In the low SE area, for some WMPF categories, unhealthy products were more expensive than healthy ones. It is possible that expenditure elasticity differed by SE area, and higher elasticity is not always expected for healthy products when healthy foods are considered a necessity and unhealthy foods a luxury [51]. In addition, higher prices for the same WMPF category in the higher SE area likely reflect higher operational costs, while larger stores such as supermarkets may also have the marketing savvy to better segment the market as compared to smaller stores. 

Although prices varied greatly across WMPF categories (as expected), higher prices for some foods also may predict lower consumption of such groups, as has been previously demonstrated in several countries [52]. While higher prices may be positive for some foods, such as the ones with added sugars as a result of the sugar tax policy, for example [53,54,55], they may have a negative impact for foods with positive ingredients and nutrients for health and nutrition outcomes [52].

### 4.5. Health and Nutrition Claims Frequent in Healthy and Unhealthy WMPFs 

The use of health and nutrition claims can inform consumers through short and clear messages about the energy and nutrient content of a product and its potential health benefits [56]. However, this strategy has been regulated in some countries due to its public health implications [57]. First, consumers perceive products displaying health and nutrition claims as healthier (health halo effect) without considering nutritional quality, and this influences their preferences and purchases [58]. Second, the regulation of these claims is limited and generally independent of nutritional quality [59]. Our results show that unhealthy WMPFs also displayed health and nutrition claims on the front-of-pack label; this suggested that consumers who make their food choices based on the information available on the label were vulnerable to misperception about the nutritional quality of these products, mainly for less-healthy groups such as breakfast cereals. Generally, WMPFs such as cookies, breads, and pastries are fortified in products that use wheat flour (mandatory regulation in Mexico). Some studies have shown the use of these practices in less-healthy foods to promote the use of health and nutrition claims [60]. Improving front-of-pack labeling regulations that include specifications for the use of health and nutrition claims contributes to helping consumers make healthy food choices, particularly in low SE areas.

### 4.6. From Traditional Healthy Foods to Unhealthy WMPFs

Some WMPF categories included in our study comprised traditional food ingredients and foods such as flour, *tortilla*, bread, and pastas; others comprised other nonbasic food commodities such as snacks and ready-to-eat foods, which have become part of the modern diet in Mexico. The WMPFs from the first categories tended to be lower in price and of better nutritional value in both SE areas. Maintaining healthy properties and ingredients of WMPFs, including dietary fiber, prebiotics [61], resistant starch content, low glycemic index, and micronutrient content, as well as reducing ingredients with a negative impact on health, is essential for WMPFs to contribute to a healthy diet, particularly among the poorer segments of the population, given that they derive a larger share of their energy from staple foods [62]. Moreover, high consumption levels of low-cost maize flour and cornmeal, especially in areas where micronutrient deficiencies exist, make this food a vehicle for fortification [63]. 

## 5. Conclusions

Healthy WMPFs have an important role to play in healthy diets [64], given their relatively low cost and concentrations of energy and essential nutrients. Transformation to healthy diets will require a greater than 50% reduction in unhealthy foods [61]. However, as highlighted in this study, the current urban retail environment is not conducive to rapid change towards greater consumption of healthy WMPFs. The extensive variety of unhealthy WMPFs available, the limited stock of healthy WMPFs in most types of retail outlets, and the confusing health and nutrition claims on packaging make it difficult to find and choose healthy WMPFs. 

The findings raise important questions about the private sector’s formulation and marketing of WMPFs and the implications for promoting healthier diets. In many developing regions, income growth will continue to push demand towards more processed foods and more foods purchased at supermarkets. The Food Systems Summit in 2021 stated that “the food choices people are faced with and the choices they make are profoundly determined by the food system of which they are part” [65], and adding other domains of the food environment to the marketing strategies used can be a win–win for the food processors, vendors, and food choices we have in Mexico City. 

This study represents a much-needed initial step to initiate dialogue between researchers, the private sector, and government agencies on how to improve access to healthier WMPFs. It highlights issues of differential access by consumers from higher and lower socioeconomic strata to healthier processed foods. 

Strengths in our study include the careful selection of the high and low socioeconomic areas (i.e., first and last deciles of the socioeconomic areas within Mexico City and surroundings) and the variety of food retail outlets visited. To the best of our knowledge, no other study has included all available food retail outlets that comply with our inclusion criteria in a given area. Future work in this area should look to engage the consumers, private sector, government agencies, and civil society on options for increasing access to and purchases of healthy WMPFs.

## Figures and Tables

**Table 1:** Distribution of healthy and unhealthy wheat and maize processed foods (WMPFs) across food retail outlets and socioeconomic areas.

Nutri-Score Profile by Retail Outlet	Unique WMPFs (*n*)	Distribution across SE Areas		
		% Low and High SE Areas	% Only Low SE Area	% Only High SE Area
Supermarkets				
Unhealthy WMPFs	1212	23.3	9.6	67.1
Healthy WMPFs	614	12.1	8.6	79.3
Total WMPFs	1826	19.5	9.3	71.2
Independently owned grocery stores				
Unhealthy WMPFs	340	72.9	6.8	20.3
Healthy WMPFs	45	68.9	6.7	24.4
Total WMPFs	385	72.5	6.7	20.8
Convenience stores				
Unhealthy WMPFs	375	62.4	7.5	30.1
Healthy WMPFs	29	51.7	3.5	44.8
Total WMPFs	404	61.6	7.2	31.2
Neighborhood corner stores				
Unhealthy WMPFs	474	67.9	19.8	12.2
Healthy WMPFs	120	47.5	37.5	15.0
Total WMPFs	594	63.8	23.4	12.8
Total				
Unhealthy WMPFs	1754	30.2	13.3	56.5
Healthy WMPFs	708	13.8	12.3	73.9
Total WMPFs	2462	25.5	13.0	38.5

**Table 2:** Distribution of WMPF categories across Nutri-Score profiles ^1^ and SE area.

Food Product Category	WMPFs (*n*)	Distribution of WMPFs		
		% Low and High SE Areas	% Only Low SE Area	% Only High SE Area
Nutri-Score Profile A ^2^				
Ready-to-eat foods	62	8.1	16.1	75.8
Breakfast cereals	11	18.2	9.1	72.7
Salty snacks	16	0.0	6.3	93.8
Cookies	19	0.0	0.0	100.0
Flour	64	28.1	18.8	53.1
Breads	146	68.5	0.0	31.5
Bars and pastries	12	0.0	0.0	100.0
Pastas	492	42.5	15.0	42.5
Maize tortillas and tortilla products	67	37.3	1.5	61.2
Total WMPFs classified	889	40.4	11.1	48.5
Nutri-Score profile B ^3^				
Ready-to-eat foods	76	57.9	5.3	36.8
Breakfast cereals	37	37.8	18.9	43.2
Salty snacks	26	0.0	3.9	96.2
Cookies	37	48.7	5.4	46.0
Flour	21	42.9	28.6	28.6
Breads	155	61.9	0.0	38.1
Bars and pastries	6	0.0	16.7	83.3
Pastas	23	0.0	0.0	100.0
Maize tortillas and tortilla products	46	54.4	13.0	32.6
Total WMPFs classified	427	48.2	6.3	45.4
Nutri-Score profile C ^4^				
Ready-to-eat foods	138	56.5	5.8	37.7
Breakfast cereals	157	43.3	5.1	51.6
Salty snacks	107	48.6	13.1	38.3
Cookies	155	47.7	1.9	50.3
Flour	96	72.9	14.6	12.5
Breads	120	61.7	1.7	36.7
Bars and pastries	45	51.1	2.2	46.7
Pastas	9	0.0	0.0	100.0
Maize tortillas and tortilla products	98	55.1	9.2	35.7
Total WMPFs classified	925	53.3	6.4	40.3
Nutri-Score profile D ^5^				
Ready-to-eat foods	213	85.5	1.4	13.2
Breakfast cereals	172	62.2	19.2	18.6
Salty snacks	1263	86.7	4.6	8.7
Cookies	585	52.5	7.7	39.8
Flour	60	43.3	15.0	41.7
Breads	80	76.3	1.3	22.5
Bars and pastries	737	83.6	1.6	14.8
Pastas	5	0.0	0.0	100.0
Maize tortillas and tortilla products	225	76.9	3.6	19.6
Total WMPFs classified	3340	76.9	5.1	18.1
Nutri-Score profile E ^6^				
Ready-to-eat foods	2	0.0	0.0	100.0
Breakfast cereals	10	70.0	0.0	30.0
Salty snacks	186	83.9	7.0	9.1
Cookies	963	72.8	3.6	23.6
Flour	48	16.7	2.1	81.3
Breads	1	0.0	0.0	100.0
Bars and pastries	769	87.7	2.0	10.4
Pastas	0	0.0	0.0	0.0
Maize tortillas and tortilla products	7	0.0	42.9	57.1
Total WMPFs classified	1986	77.8	3.4	18.8

^1^ Nutri-Score profile is a scale that divides food products into 5 classes (A to E) to distinguish healthier foods (A score) from the less healthy (E score). ^2^ Based on the Nutri-Score profile, includes all those products which had a score ≤ −1. ^3^ Based on the Nutri-Score profile, includes all those products which had a score between 0 and 2. ^4^ Based on the Nutri-Score profile, includes all those products which had a score between 3 and 10. ^5^ Based on the Nutri-Score profile, includes all those products which had a score between 11 and 18. ^6^ Based on the Nutri-Score profile, includes all those products which had a score ≥ 19.

**Table 3:** Distribution of geometric mean price (per 100 g) of WMPF categories across Nutri-Score profiles and SE areas.

Product Category	WMPFs with Healthy Nutri-Score Profile ^1^					WMPFs with Unhealthy Nutri-Score Profile ^2^				
	High SE Area		Low SE Area		GM ratio ^3^ (95%CI)	High SE Area		Low SE Area		GM Ratio ^3^ (95%CI)
	*n*	GM (95%CI)	*n*	GM (95%CI)		*n*	GM (95%CI)	*n*	GM (95%CI)	
Ready-to-eat foods	102	0.76 (0.61, 0.94)	36	0.52 (0.47, 0.57)	1.46 (1.15, 1.86)	221	0.80 (0.72, 0.88)	132	0.63 (0.55, 0.73)	1.26 (1.06, 1.49)
Breakfast cereals	33	0.60 (0.43, 0.82)	15	0.37 (0.33, 0.42)	1.59 (1.14, 2.23)	214	0.71 (0.63, 0.80)	125	0.47 (0.43, 0.52)	1.51 (1.29, 1.77)
Salty snacks	40	1.31 (1.00, 1.73)	-	-	-	823	0.97 (0.91, 1.03)	733	0.89 (0.82, 0.96)	1.09 (0.99, 1.21)
Cookies	45	1.04 (0.74, 1.45)	11	0.35 (0.33, 0.36)	3.01 (2.15, 4.21)	1112	0.84 (0.66, 1.06)	591	0.55 (0.49, 0.62)	1.53 (1.18, 1.99)
Flour	56	0.21 (0.17, 0.25)	29	0.13 (0.10, 0.17)	1.60 (1.16, 2.23)	134	0.48 (0.43, 0.53)	70	0.41 (0.36, 0.46)	1.18 (1.01, 1.38)
Breads	232	0.45 (0.36, 0.56)	69	0.35 (0.34, 0.37)	1.26 (1.00, 1.59)	145	0.51 (0.39, 0.68)	56	0.43 (0.40, 0.46)	1.20 (0.90, 1.60)
Cereal bars, sweet breads, and pastries	17	1.13 (0.88, 1.45)	-	-	-	993	0.75 (0.71, 0.80)	558	0.68 (0.65, 0.72)	1.10 (1.01, 1.19)
Pastas	334	0.35 (0.20, 0.62)	181	0.13 (0.12, 0.14)	2.66 (1.50, 4.72)	14	1.34 (0.54, 3.30)	-	-	-
Maize tortillas and tortilla products	88	0.51 (0.34, 0.75)	25	0.26 (0.21, 0.33)	1.93 (1.22, 3.05)	223	0.42 (0.35, 0.50)	107	0.31 (0.29, 0.33)	1.34 (1.11, 1.62)
Total	947	0.47 (0.34, 0.65)	366	0.20 (0.18, 0.23)	2.30 (1.60, 3.30)	3879	0.77 (0.70, 0.85)	2372	0.64 (0.59, 0.71)	1.20 (1.05, 1.37)

SE: socioeconomic. GM: geometric mean. Prices expressed in USD per 100 g. Only categories with at least 5 observations are shown. ^1^ Classification based on the combination of Nutri-Score profiles A and B. ^2^ Classification includes WMPFs with Nutri-Score profiles C, D, and E. ^3^ Geometric mean price ratio between high and low SE, reference is low SE. Estimates do not exactly match the ratios from shown geometric means because of rounding.

**Table 4:** Geometric mean price (per 100 g) for wheat and maize processed foods (WMPFs) across Nutri-Score profile in food retail outlets and by socioeconomic (SE) area.

Product Category	WMPFs with Healthy Nutri-Score Profile ^1^					WMPFs with Unhealthy Nutri-Score Profile ^2^				
	High SE Area		Low SE Area		GM Ratio ^3^	High SE Area		Low SE Area		GM Ratio ^3^
	*n*	GM (95%CI)	*n*	GM (95%CI)	(95%CI)	*n*	GM (95%CI)	*n*	GM (95%CI)	(95%CI)
**Supermarkets**										
Ready-to-eat foods	91	1.41 (0.52, 3.84)	21	0.62 (0.58, 0.66)	2.28 (0.84, 6.21)	110	1.20 (0.57, 2.54)	38	0.57 (0.56, 0.58)	2.12 (1.00, 4.46)
Breakfast cereals	27	2.62 (2.18, 3.14)	10	1.67 (1.64, 1.70)	1.57 (1.31, 1.88)	174	2.80 (2.31, 3.41)	72	1.32 (0.93, 1.86)	2.13 (1.43, 3.16)
Salty snacks	32	1.62 (1.39, 1.88)	-	-	-	222	1.45 (1.17, 1.80)	103	1.08 (0.96, 1.21)	1.35 (1.05, 1.72)
Cookies	38	2.53 (1.68, 3.80)	-	-	-	495	2.55 (1.80, 3.62)	44	0.92 (0.92, 0.92)	2.79 (1.96, 3.96)
Flour	52	1.70 (1.36, 2.13)	16	0.65 (0.24, 1.78)	2.62 (0.94, 7.35)	102	1.80 (1.33, 2.43)	29	0.94 (0.88, 1.00)	1.91 (1.41, 2.60)
Breads	106	2.19 (1.82, 2.65)	16	1.56 (1.53, 1.60)	1.40 (1.16, 1.69)	77	1.80 (1.54, 2.10)	23	1.34 (0.95, 1.87)	1.35 (0.93, 1.96)
Cereal bars, sweet breads, and pastries	9	1.57 (1.16, 2.11)	-	-	-	184	1.53 (1.32, 1.79)	56	1.13 (1.11, 1.15)	1.36 (1.17, 1.59)
Pastas	277	1.44 (0.48, 4.33)	45	0.33 (0.29, 0.39)	4.33 (1.43, 13.14)	14	2.98 (0.90, 9.87)	-	-	-
Maize tortillas and tortilla products	60	1.61 (1.02, 2.53)	8	1.04 (0.82, 1.32)	1.55 (0.93, 2.59)	94	1.24 (0.96, 1.60)	9	0.99 (0.94, 1.05)	1.25 (0.96, 1.62)
Total	692	1.67 (0.89, 3.10)	116	0.63 (0.57, 0.69)	2.65 (1.41, 4.97)	1473	1.93 (1.49, 2.49)	399	1.03 (0.95, 1.13)	1.86 (1.42, 2.44)
**Independently owned grocery stores/convenience stores**										
Ready-to-eat foods	11	0.56 (0.27, 1.17)	12	0.44 (0.33, 0.58)	1.29 (0.59, 2.84)	63	0.77 (0.60, 0.99)	50	0.51 (0.36, 0.74)	1.50 (0.96, 2.34)
Breakfast cereals	6	0.81 (0.80, 0.82)	5	1.13 (0.75, 1.71)	0.71 (0.47, 1.07)	22	1.02 (0.65, 1.59)	49	0.92 (0.64, 1.32)	1.10 (0.62, 1.96)
Salty snacks	7	0.56 (0.33, 0.94)	-	-	-	236	0.75 (0.59, 0.95)	156	0.88 (0.69, 1.12)	0.85 (0.60, 1.19)
Cookies	5	0.76 (0.63, 0.92)	-	-	-	266	0.73 (0.66, 0.80)	188	0.84 (0.75, 0.94)	0.86 (0.74, 1.00)
Flour	-	-	13	0.55 (0.32, 0.95)	-	24	0.40 (0.25, 0.63)	37	0.43 (0.33, 0.55)	0.93 (0.55, 1.59)
Breads	37	1.40 (1.27, 1.53)	11	1.35 (1.23, 1.49)	1.03 (0.90, 1.18)	27	1.31 (1.27, 1.35)	11	1.34 (1.09, 1.65)	0.98 (0.79, 1.20)
Cereal bars, sweet breads, and pastries	5	0.45 (0.32, 0.64)	-	-	-	295	0.76 (0.68, 0.86)	110	0.82 (0.73, 0.92)	0.93 (0.79, 1.09)
Pastas	20	0.30 (0.27, 0.33)	81	0.26 (0.23, 0.28)	1.17 (1.01, 1.35)	-	-	-	-	-
Maize tortillas and tortilla products	11	0.77 (0.65, 0.92)	11	0.79 (0.73, 0.86)	0.98 (0.81, 1.19)	44	1.00 (0.93, 1.07)	29	1.02 (0.88, 1.18)	0.98 (0.84, 1.16)
Total	102	0.73 (0.54, 1.01)		0.39 (0.31, 0.48)	1.90 (1.29, 2.79)	977	0.76 (0.68, 0.86)	630	0.80 (0.73, 0.88)	0.95 (0.81, 1.11)
**Neighborhood corner store**										
Ready-to-eat foods	-	-	-	-	-	48	0.53 (0.48, 0.57)	44	0.50 (0.48, 0.52)	1.05 (0.97, 1.15)
Breakfast cereals	-	-	-	-	-	18	0.55 (0.41, 0.75)	-	-	-
Salty snacks	-	-	-	-	-	365	0.52 (0.50, 0.54)	474	0.51 (0.50, 0.52)	1.03 (0.99, 1.07)
Cookies	-	-	6	0.64 (0.62, 0.66)	-	351	0.61 (0.60, 0.62)	359	0.65 (0.63, 0.67)	0.94 (0.90, 0.98)
Flour	-	-	-	-	-	8	0.52 (0.42, 0.65)	-	-	-
Breads	89	1.40 (1.32, 1.49)	42	1.23 (1.11, 1.35)	1.15 (1.02, 1.28)	41	1.30 (1.17, 1.45)	22	1.01 (0.95, 1.08)	1.29 (1.13, 1.46)
Cereal bars, sweet breads, and pastries	-	-	-	-	-	514	0.64 (0.62, 0.67)	392	0.64 (0.61, 0.67)	1.01 (0.94, 1.08)
Pastas	37	0.32 (0.31, 0.33)	55	0.32 (0.31, 0.33)	1.01 (0.96, 1.05)	-	-	-	-	-
Maize tortillas and tortilla products	17	0.88 (0.78, 0.98)	6	0.81 (0.79, 0.83)	1.08 (0.96, 1.22)	84	1.03 (0.97, 1.09)	44	0.94 (0.88, 1.00)	1.10 (1.01, 1.19)
Total	143	0.91 (0.75, 1.09)	109	0.58 (0.47, 0.72)	1.55 (1.17, 2.05)	1429	0.63 (0.61, 0.64)	1335	0.60 (0.59, 0.61)	1.04 (1.02, 1.07)

SE: socioeconomic. GM: geometric mean. Prices expressed in USD per package. Only categories with at least 5 observations are shown. ^1^ Classification based on the combination of Nutri-Score profiles A and B. ^2^ Classification based on the combination of Nutri-Score profiles C, D, and E. ^3^ Geometric mean price ratio between high and low SE, reference is low SE. Estimates do not exactly match the ratios from shown geometric means because of rounding.

**Table 5:** Difference in the percentage of healthy and unhealthy wheat and maize processed foods (WMPFs) displaying health and nutrition claims on the front of the package by socioeconomic (SE) area.

Product Category	WMPFs with Healthy Nutri-Score Profile ^1^					WMPFs with Unhealthy Nutri-Score Profile ^2^				
	High SE Area		Low SE Area		Difference (95%CI) ^3^	High SE Area		Low SE Area		Difference (95%CI) ^3^
	*n*	% (95%CI)	*n*	% (95%CI)		*n*	% (95%CI)	*n*	% (95%CI)	
Ready-to-eat foods	102	21.6 (17.1, 26.9)	36	19.4 (10.9, 32.4)	2.1 (−9.7, 13.9)	221	10.0 (5.5, 17.2)	132	2.3 (0.4, 11.1)	7.7 (0.9, 14.5)
Breakfast cereals	33	66.7 (52.8, 78.1)	15	66.7 (54.9, 76.6)	0 (−16.9, 16.9)	214	53.7 (47.5, 59.8)	125	64.8 (57.1, 71.8)	−11.1 (−20.7, −1.4)
Salty snacks	40	30.0 (11.3, 59.0)	-	-	-	823	7.4 (4.2, 12.8)	733	3.3 (2.4, 4.4)	4.1 (−0.1, 8.4)
Cookies	45	51.1 (37.9, 64.1)	11	18.2 (4.0, 54.4)	32.9 (4.6, 61.3)	1112	15.1 (9.6, 23.0)	591	7.4 (5.8, 9.4)	7.7 (0.8, 14.5)
Flour	56	62.5 (52.7, 71.3)	29	55.2 (37.4, 71.7)	7.3 (−14.8, 29.4)	134	36.6 (28.1, 45.9)	70	40.0 (29.1, 51.9)	−3.4 (−18.1, 11.2)
Breads	232	57.8 (48.3, 66.7)	69	53.6 (45.7, 61.4)	4.1 (−12.8, 27.5)	145	37.2 (30.0, 45.1)	56	17.9 (11.0, 27.8)	19.4 (8.0, 30.7)
Cereal bars, sweet breads, and pastries	17	23.5 (2.9, 76.1)	-	-	-	993	22.2 (18.2, 26.7)	558	17.4 (12.9, 23.0)	4.8 (−1.8, 11.4)
Pastas	334	43.4 (30.9, 56.8)	181	70.2 (55.4, 81.7)	−26.8 (−45.6, −7.9)	14	7.1 (0.7, 44.0)	-	-	-
Maize tortillas and tortilla products	88	72.7 (63.0, 80.7)	25	40.0 (21.7, 61.6)	32.7 (9.9, 55.6)	223	40.4 (30.9, 50.5)	107	18.7 (11.1, 29.7)	21.7 (8.1, 35.2)
Total WMPFs	947	48.7 (41.1, 56.4)	366	56.6 (46.3, 66.5)	−7.9 (−20.8, 4.9)	3879	20.1 (14.8, 26.5)	2372	12.9 (9.2, 17.9)	7.2 (−0.1, 14.4)

SE: socioeconomic. GM: geometric mean. Prices expressed in USD per package. Only categories with at least 5 observations are shown. ^1^ Classification based on the combination of Nutri-Score profiles A and B. ^2^ Classification based on the combination of Nutri-Score profiles C, D, and E. ^3^ Difference of percentage of products displaying health and nutrition claims on the front of the package in high SE areas with respect to low SE areas (percentage points).

## Data Availability

The data presented in this study are available on request from the corresponding author. The data are not publicly available due to the research team continuing with further analyses of this study.
